# Phosphorylation of Nonmuscle myosin II-A regulatory light chain resists Sendai virus fusion with host cells

**DOI:** 10.1038/srep10395

**Published:** 2015-05-20

**Authors:** Provas Das, Shekhar Saha, Sunandini Chandra, Alakesh Das, Sumit K. Dey, Mahua R. Das, Shamik Sen, Debi P. Sarkar, Siddhartha S. Jana

**Affiliations:** 1 Department of Biological Chemistry, Indian Association for the Cultivation of Science, Jadavpur, Kolkata-700032; 2 Department of Biochemistry, University of Delhi South Campus, New Delhi-1100021; 3 Department of Biosciences and Bioengineering, Indian Institute of Technology-Bombay, Mumbai-400076

## Abstract

Enveloped viruses enter host cells through membrane fusion and the cells in turn alter their shape to accommodate components of the virus. However, the role of nonmuscle myosin II of the actomyosin complex of host cells in membrane fusion is yet to be understood. Herein, we show that both (−) blebbistatin, a specific inhibitor of nonmuscle myosin II (NMII) and small interfering RNA markedly augment fusion of Sendai virus (SeV), with chinese hamster ovary cells and human hepatocarcinoma cells. Inhibition of RLC phosphorylation using inhibitors against ROCK, but not PKC and MRCK, or overexpression of phospho-dead mutant of RLC enhances membrane fusion. SeV infection increases cellular stiffness and myosin light chain phosphorylation at two hour post infection. Taken together, the present investigation strongly indicates that Rho-ROCK-NMII contractility signaling pathway may provide a physical barrier to host cells against viral fusion.

Sendai virus (SeV), an enveloped animal virus of the family Paramyxoviridae, is well characterized and known to enter its host cells by membrane fusion^1^,^2^,^3^. This virus has also been widely used to study the stages of the Paramyxoviridae life cycle comprising viral binding, fusion and subsequent egress. The infection process is initiated by the binding of the virus to sialic acid of host cells by viral hemagglutinin-neuraminidase (HN glycoprotein) followed by fusion of the viral membrane with the host plasma membrane mediated by a fusion protein, F glycoprotein^4^,^5^. The latter finally culminates in generation of a fusion pore through which viral nucleocapsid is delivered into the host cells, leading to a complete infection process. Following such successful fusion events, the appearance of viral HN and F proteins on the surface of the host plasma membrane catalyzes its fusion with adjacent cell membranes resulting in multinucleated giant cells^6^. Although the role of HN and F in fusion mediated viral entry is well established, little is known about the influence of many host proteins, especially those that are involved in non-viral fusion events like myotube formation, exocytosis, endocytosis, and phagocytosis^7^,^8^,^9^,^10^. The crucial step in the process of myotube formation is membrane fusion which is known to be primarily controlled by nonmuscle myosin II (NMII) proteins^11^,^12^. But, little is known about the functional importance of NMII in virus-cell fusion.

NMIIs are molecular motors that bind to filamentous actin in an ATP-dependent manner and are also known as mechanoenzymes, as they translocate actin filaments using energy released from ATP hydrolysis^13^. NMII is a hexameric protein composed of one pair each of heavy chain of molecular mass of 220 kDa, essential light chain (ELC) of 17 kDa and regulatory light chain (RLC) of 20 kDa. RLC phosphorylation promotes the formation of myosin minifilaments that slide along the actin filament in opposite direction using energy released from its actin-dependent ATP hydrolysis activity^14^. In humans, three isoforms of nonmuscle myosin heavy chain (NMHC) II, termed NMHC II-A, II-B and II-C, are encoded by three different genes^14^. They play important roles in cellular functions such as cytokinesis, migration, and adhesion^15^,^16^. However, their role in viral infection is yet to be explored. Here we investigate the participation of NMII isoforms of host cells in regulating viral fusion.

## Results

### Perturbation of NMII activity increases membrane fusion and virion release

**Figure 1:**
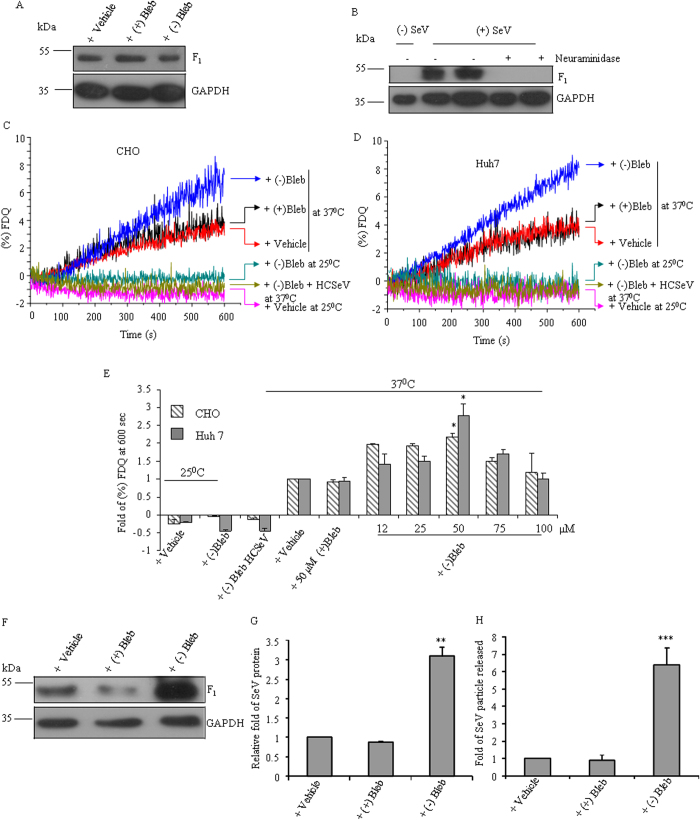
Perturbation of NMII activity increases membrane fusion. (**A**) (−) blebbistatin, (+) blebbistatin or vehicle treated CHO cells were allowed to bind with SeV at 4 °C for 1 h. After removing unbound SeV, lysates were prepared and probed with anti-SeV antibody. Only the band near 55 kDa, which is F_1_ fragment of F protein, is shown here. (**B**) SeV bound CHO cells were treated with or without neuraminidase for 1 h at 37 °C. Two different amounts, 10 and 20 μg of cell lysates *(+SeV)* were probed with anti-SeV antibody. Noninfected CHO cells *(-SeV)* were considered as negative control. (**C-D**) R18 labeled bound SeV was allowed to fuse at 37 °C with drug or vehicle treated CHO (**C**) and Huh 7 cells (**D**). R18 dequenching due to membrane mixing was recorded online and (%) FDQ was plotted against time. (**E**) Fold change in % FDQ was quantified at 600 sec in all events, considering vehicle treated CHO and Huh7 cells at 37 °C as “1”. Virus-cell fusion exhibited a dose dependent relationship in respect to (−) blebbistatin. *, p < 0.05 for vehicle vs. 50 μM (−) blebbistatin treated CHO or Huh7 cells at 37 °C, Results are expressed as mean ± SEM, n = 3. FDQ, fluorescence dequenching; HCSeV, heat inactivated Sendai virus. (**F**) Cell lysates of 24 hpi CHO cells that were pre-treated with (−) blebbistatin, (+) blebbistatin or vehicle were subjected to immunoblot analysis for SeV proteins. (**G**) Immunoblots shown in F were quantified using ImageJ software. One representative set of blots from three independent experiments is shown here. (**H**) Hemagglutinin activity was measured with culture media of 48 hpi CHO cells that were pre-treated with (−) blebbistatin, (+) blabbistatin or vehicle. Fold change was calculated using a formula; fold = 2^N^/2^n^ = 2^N−n^, where N and n stand for the well number for red blood cell agglutination of culture medium from drug and vehicle treated cells, respectively. For all immunoblots, GAPDH was used as loading control. Results are expressed as mean ± SEM, n = 5. *, p < 0.05; **, p < 0.01; ***, ‘p < 0.001 for vehicle vs (−) blebbistatin treated CHO cells. hpi, hours post infection.

To investigate the role of NMII in infection of CHO cells by SeV (binding, fusion and budding of progeny viral particles from host cells) we, first, interfered with NMII activity by treating CHO cells with (−) blebbistatin^17^,^18^. The amount of virus bound to CHO cells after 1 h incubation was monitored and Fig. 1A shows that binding of the virus to the cells is not affected in presence of (−) blebbistatin or its inactive enantiomer (+) blebbistatin when compared with vehicle, as marked by the band below 55 kDa (corresponding to the F_1_ fragment of F protein).

To make sure that virus binding is through interaction of HN protein with cells’ sialic acid containing receptor we treated the cells with neuraminidase after binding to SeV. Figure 1B shows no binding of SeV in the presence of neuraminidase. After binding, cells were allowed to fuse with R18 labeled SeV at 37 °C and kinetics of lipid mixing during membrane fusion were measured over 600 sec. When the viral and host plasma membranes get fused, the R18 molecules gets spontaneously transferred to the host cell membrane and diffuse rapidly (mainly lateral). Such event releases the self quenching of R18 molecules culminating into enhancement (dequenching) of R18 fluorescence (FDQ). Percentage of FDQ is calculated and it is directly proportional to the membrane fusion. Figure 1C,D show at 600 sec, more than two fold fusogenic activity was achieved with 50 μM (−) blebbistatin treated CHO cells or Huh7 cells as compared with vehicle or (+) blebbistatin, suggesting the possibility of involvement of NMII activity in the membrane fusion. Interestingly, no fusion was observed when heat-inactivated virion particles were used for the kinetic study or with active virions at 25 °C with 50 μM (−) blebbistatin treated CHO and Huh7 cells, suggesting that virus-cell membrane fusion is also dependent on the activity of NMIIs of host cells other than physiological temperature and activity of viral proteins. Virus-cell fusion exhibited a dose dependent relationship in respect to (−) blebbistatin (Fig.1E). As the cells started fragmentation in the presence of 75 μM (−) blebbistatin and above, we treated CHO and Huh7 cells with 50 μM (−) blebbistatin in our subsequent experiments. With a view to critically assess complete fusion (membrane mixing and cytoplasmic continuity), a cell-cell fusion assay was carried out as reported earlier^19^. Supplementary Fig. 1A-B shows that (−) blebbistatin induces significant core mixing (more than four fold) as visualized by the appearance of yellow color in merged panels. Increase in membrane mixing was also visualized by time lapse microscopy in SeV infected CHO cells treated with (−) blebbistatin as compared with vehicle (Supplementary movie 1,2).

Moreover, an increase in membrane fusion event is expected to augment the production and release of viral particles from host cells. This is, in fact, supported by a sharp increase (2.7 ± 0.9 fold) in the amount of viral protein F_1,_ (Fig. 1F,G) in the infected cells and enhanced (6.2 ± 0.8 fold) release of progeny virion particles (Fig. 1H). Taken together, these data suggest that membrane fusion and virion release, but not virion binding, are affected by NMII activity.

### Effect of siRNA against NMII in virus-cell fusion

**Figure 2:**
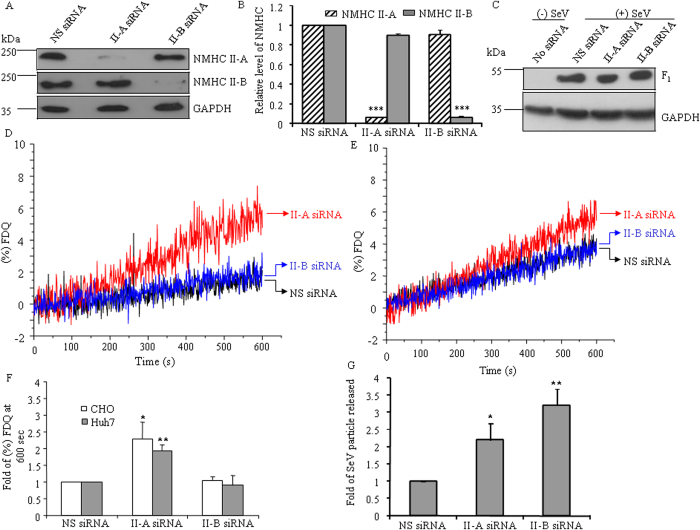
SiRNA against NMII-A in CHO cells increases fusion and virion release. (**A**) Lysates of 72 h post siRNA treated CHO cells were probed with NMHC II isoform specific antibodies (upper part of the membrane), as indicated. GAPDH (lower part of the membrane) was used as a loading control for cell lysates. (**B**) Immunoblots were quantified using ImageJ software. Fold change was calculated considering relative band intensity of each isoform in nonspecific siRNA treated sample as “1”. ***, p < 0.001 for NMHC IIA in NS siRNA vs. II-A siRNA treated CHO cells, and for NMHC II-B in NS siRNA vs. II-B siRNA treated CHO cells. Note that NMHC II-A was almost unaltered by II-B siRNA and vice versa. (**C**) SiRNA treated CHO cells were allowed to bind SeV at 4 °C for 1 h. After removing unbound SeV, lysates were prepared and immunoblots were probed with antibody against SV proteins. Blot was stripped and reprobed with GAPDH antibody. (**D-E**) Fusion kinetics of membrane mixing were performed between CHO (**D**) or Huh7 (**E**) cells that had been previously treated with siRNA for 72 h and R18 labeled SeV and monitored until 600 sec. (**F**) Fold change in % FDQ was quantified at 600 sec in all fusion reactions considering nonspecific siRNA treated CHO and Huh7 cells at 37 °C as “1”. *, p < 0.05 for NS siRNA vs. II-A siRNA in CHO cells and **, p < 0.01 for NS siRNA vs. II-A siRNA in Huh 7 cells. Results are expressed as mean ± SEM from three independent experiments. FDQ, fluorescence dequenching. (**G**) Hemagglutinin activity was carried out with culture media of 48 hpi CHO cells that were previously treated with siRNA for 72 h. Fold change of virion particles released was calculated by dividing the hemagglutinin activity value of culture media from nonspecific siRNA treated CHO cell. *, p < 0.05 for NS siRNA vs. II-A siRNA, **, p < 0.01 for NS siRNA vs. II-B siRNA treated CHO cells. Results are expressed as mean ± SEM from five independent experiments.

In order to investigate the role of each isoform of NMII, first the relative amount of the individual isoforms of NMII in CHO cells was evaluated using mass spectroscopy analysis. We found the presence of 53 ± 3.02% II-A, 44.8 ± 2% II-B and 2.2 ± 1.2% II-C of total NMHC IIs. Such a low amount of NMII-C was undetectable by western blot and reverse transcriptase-PCR analysis (data not shown). To explore further the role of individual isoforms in viral infection, we inhibited the expression of each isoform using small interfering RNA. Figure 2A,B show that siRNA against NMHC II-A mRNA specifically inhibits the expression of NMHC II-A, but not NMHC II-B, by >90% compared to nonspecific siRNA. Similarly, siRNA against NMHC II-B mRNA specifically inhibits the expression of NMHC II-B by >92%, but not NMHC II-A. Inhibition of NMIIs expression by specific siRNA gives insignificant interference in viral binding as shown in Fig. 2C. In contrast, reduction of NMII-A, but not II-B, increases lipid mixing (2.2 ± 0.51 fold, Fig. 2D,F). Similarly, inhibition of NMII-A in Huh7 cells increases lipid mixing with SeV (1.94 ± 0.18 fold, Fig. 2E,F). On the other hand, reduction of NMII-B has a major effect on viral release (3.2 ± 0.49 fold) as revealed by the hemagglutinin assay (Fig. 2G). This study implicates the importance of NMII-A and II-B in various stages of virus life cycle.

### NMII-RLC phosphorylation during virus-host interaction

**Figure 3:**
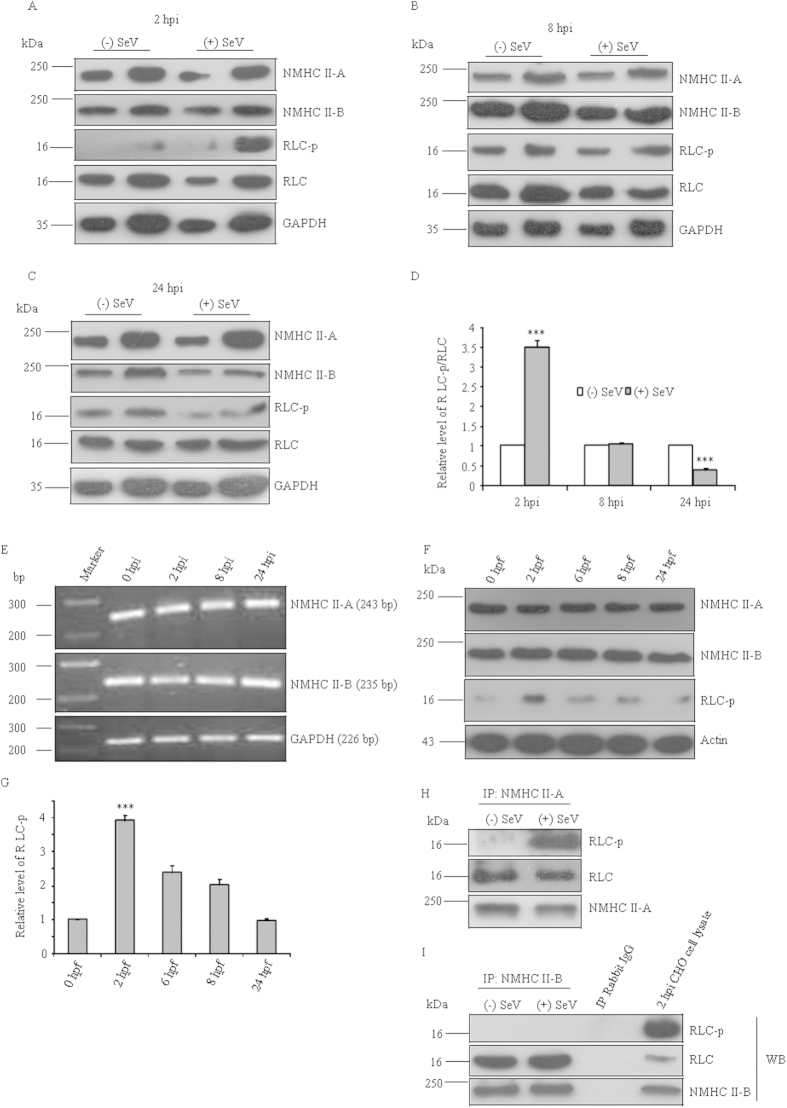
Phosphorylation of NMII-A regulatory light chain during viral life cycle. (**A-C**) 10 and 20 μg of cell lysate from CHO cells infected with SeV for 2-24 h were analyzed by immunoblot *(+SeV).* Noninfected CHO cell lysates were considered as vehicle controls *(-SeV)*. The upper part of the membranes was probed with antibody to NMHC II-A or II-B and the lower part was probed with antibody to GAPDH, phospho Ser-19 RLC, or RLC. Samples were run in parallel under same condition and a representative blot is shown here. (**D**) Quantification of immunoblots in A-C was carried out using ImageJ software. Band intensity from the RLC-p blot was normalized to RLC. Relative band intensity of RLC-p with respect to RLC was quantified considering the value of noninfected CHO cells as ‘1’. ***, p < 0.001 for (−) SeV vs (+) SeV infected CHO cells at 2 and 24 hpi. Results are expressed as mean ± SEM from three independent experiments. (**E**) Total RNA was isolated from CHO cells infected with SeV for 0-24 h, as indicated and RT-PCR analysis was performed using primers specific for NMHC II-A (upper panel), -II-B (middle panel), or GAPDH (lower panel). (**F**) Lysates of Huh7 cells fused with F-HN VLP (devoid of viral genome) for different time period, as indicated, were separated on SDS-PAGE and probed with anti-NMHC II-A or II-B (upper part of the membrane), and RLC-p or Actin (lower part of the membrane) antibodies. (**G**) Quantification of immunoblots. Relative fold induction of RLC-p was calculated by considering relative band intensity of RLC-p in noninfected Huh7 cells as “1”. ***, p < 0.001 for 0 hpf vs. 2 hpf. n = 3. (H-I) At 2 hpi, cell lysate was used for immunoprecipitation (IP) with NMHC II-A (**H**) or –II-B (**I**) specific antibody. Immunoprecipitates using NMHC II antibodies were subjected to western blot with RLC, RLC-p, or NMHC II isoform specific antibody. Rabbit IgG was used as negative control for immunoprecipitation. The experiment was repeated three times. hpi, hours post infection; hpf, hours post fusion; 0 hpi indicates no infection, 0 hpf indicates no fusion; (−) SeV, without SeV; (+SeV), with SeV.

In order to gain molecular insight into the regulation of virus-host interaction by NMII isoform(s), a systematic study involving the expression of NMII-A and II-B in host cells was carried out in a time dependent manner and correlated with SeV infection. Figure 3A–C,E show that expression of NMHC II-A and II-B both at the protein and mRNA levels remain unchanged at various times of infection, but phosphorylation of RLC is significantly increased by 3.5 ± 0.16 fold at 2 hpi compared with noninfected CHO cells. At 8 hpi, the level of phosphorylation remains same whereas at 24 hpi, it is decreased to almost 40% of that of noninfected cells. Note that phosphorylation of RLC is increased in cells without viral infection which could be due to cell spreading^20^. This intrinsic increase of phosphorylation may add to the extrinsic increase of phosphorylation of RLC of host cells with viral infection. However, our observation stands significant because calculation of the fold difference was done with respect to noninfected cells for each time point. To eliminate any role of the virus genome in changing NMII activity, we performed the experiment with F,HN-virosome (devoid of the viral genome and its associated viral proteins, called virus like particle, VLP) and Huh7 cells. Figure 3F shows the F,HN-VLP does not change the expression of NMHC II through 24 hpf, but induces phosphorylation of RLC at 2 hpf as observed in the case of intact virus (Fig. 3G,D), suggesting that the viral genome is less likely to modulate NMII activity.

A recent report reveals that NMIIs have different RLC isoforms, implying that NMHC II-A and –II-B can bind to different light chains^21^. To ascertain the isoform specific light chain phosphorylation, we immunoprecipitated each NMII isoform from CHO cell extracts at 2 hpi using NMHC II specific antibodies, and checked for RLC phosphorylation in the immunoprecipitate using Ser-19 phospho specific antibody. Figure 3H shows that the immunoprecipitate of NMHC II-A contains increased amount of Ser-19 phospho RLC in SeV infected CHO cells when compared with control. Surprisingly, under similar conditions, an immunoprecipitate of NMHC II-B did not contain detectable amount of phospho RLC (Fig. 3I). These results suggest that the expression of NMII-A or II-B remains unaltered, and RLC of only NMII-A is phosphorylated during viral fusion.

### Localization of NMII-A and II-B during viral infection

**Figure 4:**
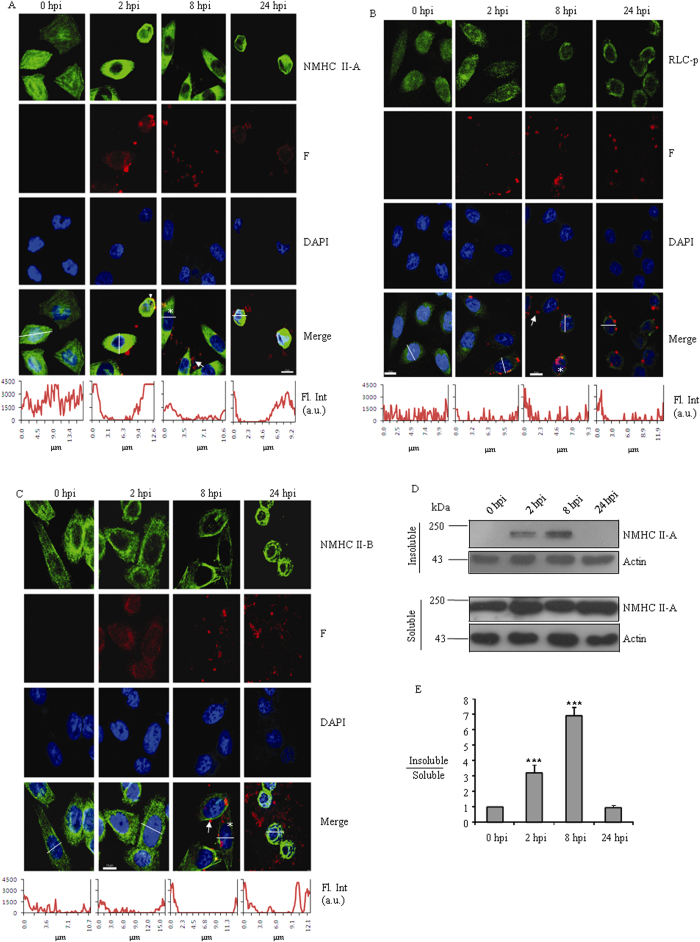
Localization of NMIIs during viral life cycle. (**A-C**), CHO cells infected with SeV for different time points, as indicated, were stained with antibody to NMHC II-A (**A**, green), phospho Ser-19 RLC (**B**, green), or NMHC II-B (**C**, green), Cells were counterstained with antibody against F protein (red) and DAPI to visualize viral protein and nucleus, respectively. Alexa-488 conjugated rabbit IgG and Alexa-594 conjugated mouse IgG were used as secondary antibodies. Scale bar - 10 μm; arrows and stars indicate viral particles outside and inside the cell, respectively; arrowhead in (**A**) shows an internalization event of viral particle at the cortex of CHO cells at 2 hpi. Quantification of fluorescence intensity (n ≥ 10 cells) at each time point of labeled cells was carried out using line scan analysis. A representative graph (across the white line) at each time point is shown in the bottom panel. (**D**) Triton X-100 insoluble (upper two panels) and soluble CHO lysates (lower two panels) were subjected to immunoblot with antibodies specific to NMHC II-A (upper part of the membrane) and Actin (lower part of the membrane). (**E**) Quantification of immunoblots was carried out using ImageJ software. Ratio of relative band intensity of insoluble to soluble lysate was calculated considering the value from 0 hpi CHO cells as ‘1’. ***, p < 0.001 for 0 hpi vs. 2 hpi or 8 hpi CHO cells. Data are expressed as mean ± SEM from three independent experiments. hpi, hours post infection; 0 hpi indicates no infection; Fl. Int, Fluorescence intensity; a.u., arbitrary unit.

Time dependent regulation of NMII RLC phosphorylation prompted us to study the localization of NMIIs during viral infection in CHO cells. Figure 4A shows that NMII-A (green) localizes at stress fibres throughout the noninfected CHO cells (0 hpi). At 2 hpi, NMII-A localizes to the cytosol and is also enriched at the cortex. An arrowhead indicates yellow color which may be due to the internalization of SeV particles (fusion) at cortex where NMII-A is enriched (Supplementary Fig. 2). We also checked RLC-p distribution at this time point (Fig. 4B) and found it to be more concentrated at the cortex. Interestingly, at 8 hpi, NMII-A is homogenously distributed throughout the cells- cytosol to cortex. Red puncta inside the cells may the synthesized new viral proteins (marked with star) and outside cells could be new virions or membranes of disrupted cells with many viral particles (marked with arrow). At 24 hpi, the majority of virion particles are outside the cells and cells are rounded. In contrast, NMII-B is distributed throughout the cell and maintains its position in stress fibres through the viral life cycle except at 24 hpi (Fig. 4C). We quantified the fluorescence intensity of Alexa 488 across an axis (one end to another) of the cells (n ≥ 10 cells at each time point of each group). This analysis suggests that viral infection leads to an increase in a wider cortex localization of NMII-A as compared with that of NMII-B. This observation suggests that the NMII-A of the host cell predominantly undergoes changes in localization during viral life cycle.

We quantified the fraction of NMII-A molecules in insoluble form (stress fibres) during viral life cycle. At various time points post infection, cell membranes were solubilized with mild Triton X-100 treatment. Soluble and insoluble fractions of Triton X-100 treated cell lysates were separated on SDS-PAGE and probed with antibody against NMHC II-A and actin. Figure 4D,E show that both at 2 and 8 hpi, the insoluble fraction (indicative of more filaments with actin) contains more NMII-A compared with either noninfected or cells at 24 hpi.

### Rho-kinase phosphorylates RLC during membrane fusion

**Figure 5:**
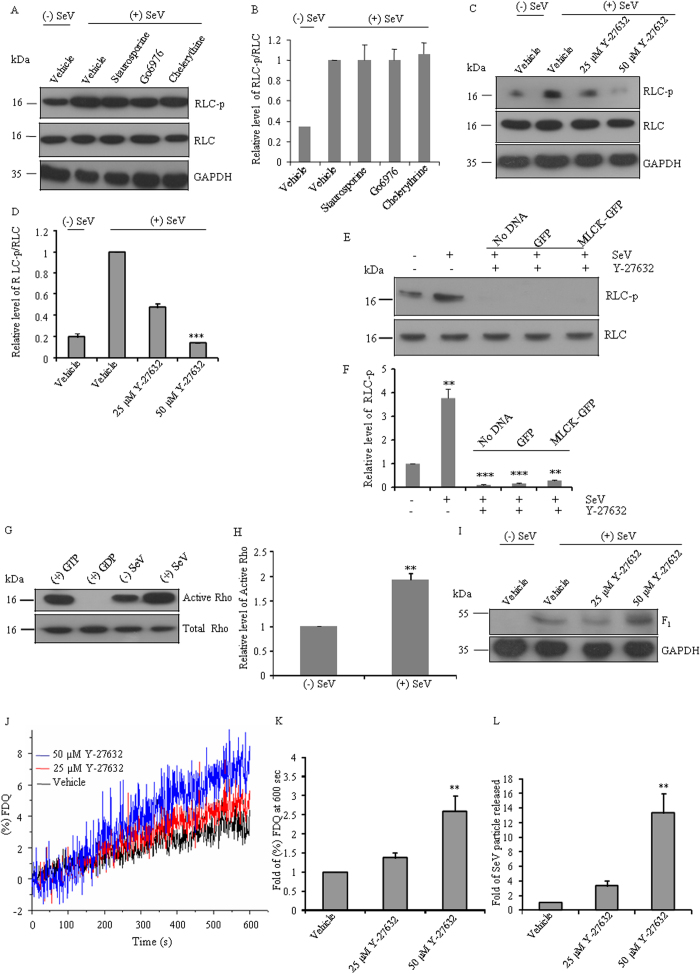
Rho kinase inhibition increases virus-cell membrane fusion and virion release. (**A**) Lysates of 2 hpi CHO cells pre-treated with or without 100 nM staurosporine, 2 μM chelerythine or 10 μM Go6976 were subjected to immunoblot using antibodies specific to RLC, RLC-p or GAPDH. Note that inhibitors are unable to reduce the SeV induced increase level of RLC-p. (**B**) Quantification of immunoblots shown in A. (**C**) Lysates of 2 hpi CHO cells pre-treated with or without Y-27632 were subjected to immunoblot using antibodies specific to RLC, RLC-p or GAPDH. (**D**) Quantification of immunoblots shown in C. (**E**) Lysates of 2 hpi Y-27632 treated CHO cells that were previously transfected with plasmid DNA encoding GFP tagged MLCK or GFP alone were subjected to immunoblot using antibodies specific to RLC, RLC-p. Mock transfection was used as control. Overexpression of MLCK could not rescue Y-27632 mediated reduction of RLC-p in SeV infected cells. **p < 0.01 for noninfected CHO cells vs MLCK expressed infected CHO cells pre-treated with Y-27632 (**F**) Quantification of immunoblots shown in E. (**G**), Lysate of 2 hpi CHO cells were used for active Rho precipitation followed by immunoblot using antibody specific for Rho (upper panel). Crude cell lysates were used to detect total Rho (lower panel). Normal CHO cell lysate (without infection) treated with GTP or GDP were used as appropriate controls for active Rho precipitation assay. (**H**) Quantification of immunoblots shown in G. (**I**) Y-27632 or vehicle treated CHO cells were allowed to bind with SeV at 4 °C for 1 h. Cell lysates were prepared and probed with anti-SeV antibody. (**J**) Bound SeV was allowed to fuse and R18 dequenching was plotted against time until 600 sec. Quantification of fold (%) FDQ at 600 sec (**K**) and hemagglutinin assay (**L**). RLC-p and SeV blots were stripped and reprobed with RLC and GAPDH antibody respectively in (**A**, **C**, **E** and **I**). **p < 0.01 for vehicle vs 50 μM Y-27632. Note that Y-27632 reduces phosphorylation of RLC in CHO cells infected with SeV for 2 h. ***p < 0.001 for vehicle vs 50 μM Y-27632. The experiment was repeated three times.

Recent studies suggest that NMII is regulated by the phosphorylation of its RLC at a conserved serine residue (Ser-19) by Protein Kinase C (PKC), myotonic dystrophy kinase-related Cdc42-binding kinase (MRCK), Rho kinase, and myosin light chain kinase (MLCK)^22^,^23^,^24^. To dissect the kinases involved in the phosphorylation of RLC, inhibitors, staurosporine and Go6976 for PKC and chelerythine for MRCK treated CHO cells were infected with SeV. Figure 5A,B show that all the inhibitors were unable to reduce the phosphorylation level of RLC at 2 hpi CHO cells. In contrast, treatment with Y-27632, an inhibitor of ROCK, leads to a significant decrease in phosphorylation of RLC at 2 hpi (Fig. 5C,D) suggesting that ROCK, but not PKC, or MRCK, may be involved in SeV induced RLC phosphorylation in host cells. When we expressed GFP tagged MLCK in CHO cells, we found an increase in phosphorylation of RLC (Supplementary Fig. 3). However, treatment of Y-27632 in SeV treated CHO cells expressing GFP tagged MLCK significantly reduces the phosphorylation of RLC (Fig. 5E,F), suggesting that MLCK is less likely to be involved in SeV induced RLC phosphorylation.

To investigate whether Rho is activated in SeV treated CHO cells, we used a Rho affinity precipitation assay, in which GST bound Rho binding domain (RBD) of the effector rhotekin pulls down active Rho[GTP]^25^. Figure 5G,H show that infected CHO cells contains 1.9 ± 0.13 fold more active Rho[GTP] compared with noninfected CHO cells at 2 hpi. This finding suggests that Rho is activated upon viral infection.

We further carried out viral binding, fusion kinetics and HAU test in presence of Y-27632. Figure 5I shows that virus binding for 1 h is slightly higher in cells treated with Y-27632 when compared with vehicle treated CHO cells. However, we found that Y-27632 could significantly increase virus host fusion (Fig. 5J,K), and viral release (Fig. 5L) in a dose dependent manner, suggesting that phosphorylation of RLC by Rho-ROCK may play an important role in viral fusion and its release.

### Effect of mutant RLCs on viral infection

**Figure 6:**
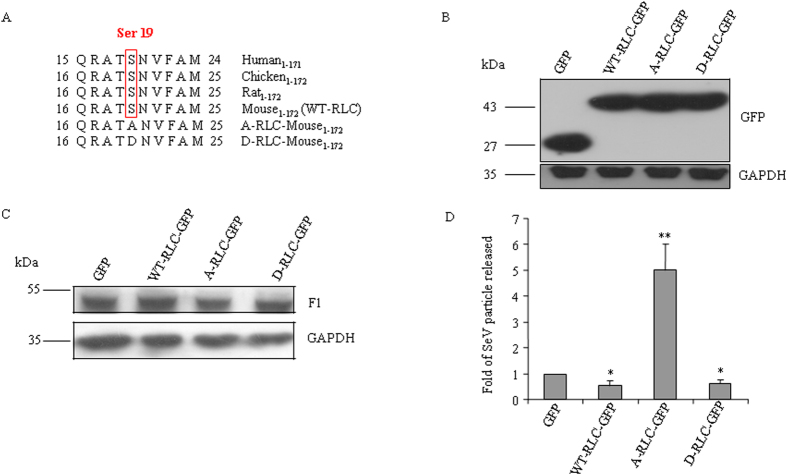
Phosphomimicking form of RLC reduces viral infection. (**A**) N-terminal amino acid sequence of mouse wildtype and mutant RLCs. Box depicts the conserved serine residue (Ser-19 for human, Ser-20 for mouse) and this residue has been mutated to Alanine (A-RLC) or Aspartic acid (D-RLC). (**B**) Lysates of CHO cells transfected with plasmid DNA containing WT-RLC-GFP, A-RLC-GFP, D-RLC-GFP or GFP were probed with antibody against GAPDH or GFP. Samples were run in two parallel gels under the same experimental condition, one for each antibody. (**C**) Plasmid DNA transfected CHO cells were allowed to bind with SeV at 4 °C for 1 h. After removing unbound SeV, cell lysates were prepared and probed with antibody against SeV proteins. Blot was stripped and reprobed with GAPDH. (**D**) Hemagglutinin activity was measured with culture media of 48 hpi CHO cells that were transfected with WT-RLC-GFP, A-RLC-GFP, D-RLC-GFP or GFP. Fold change of virion particles released was calculated by dividing the hemagglutinin activity of culture media from GFP transfected CHO cell. Results are expressed as mean ± SEM from three independent experiments. *, p < 0.05, **, p < 0.01 for GFP vs RLC-GFPs (wildtype or mutant) treated CHO cells.

To investigate the role of phosphorylated RLC in viral infection, GFP-tagged mouse mutant RLCs (S replaced with A or D) were generated as shown in Fig. 6A and introduced into CHO cells (Fig. 6B). SeV was then infected in CHO cells expressing mutants or WT-RLC. We checked the amount of virus bound to CHO cells after 1 h incubation. Figure 6C shows that the binding of virus is not altered in CHO cells expressing wildtype or mutant RLC when compared with control cells expressing only GFP. At 48 hpi, SeV particles were harvested in culture medium and hemagglutinin assay was carried out. Figure 6D shows fold of SeV particles released in culture medium of the cells that were transfected with GFP-tagged wildtype or mutant RLCs. Cells transfected with WT-RLC-GFP or phosphomimicking mutant (D-RLC-GFP) released two fold less amount of viral particles as did control cells which were transfected with GFP alone. In contrast, cells transfected with phospho-dead mutant (A-RLC-GFP) released four fold more amount of viral particles compared with control cells in culture medium. These results suggest that phosphorylation of RLC at Ser-19 decreases viral infection.

### Sendai virus increases cellular stiffness

**Figure 7:**
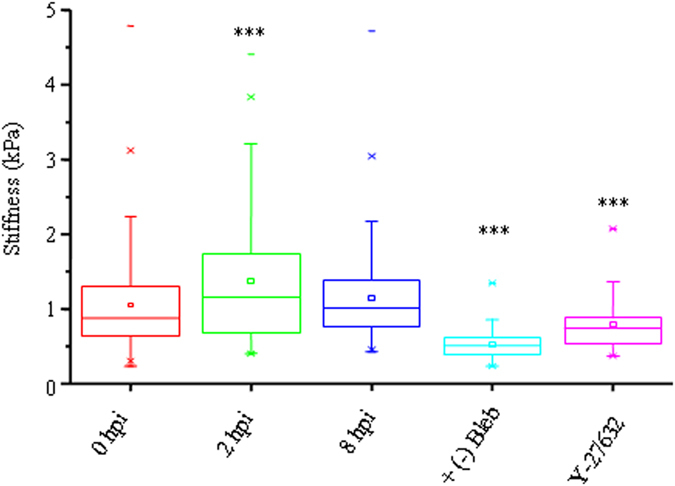
Influence of SeV infection on cortical stiffness. Cellular stiffness of SeV infected or drug treated CHO cells was measured using atomic force microscopy. Stiffness values are shown in box plot. At 2 hpi, note that 75% of infected cells exhibit more than 1.5 kPa siffness compared with that of non-infected cells, which exhibit approximately 1 kPa. ***p < 0.001 for 0 hpi vs 2 hpi. Stiffness of CHO cells in presence of myosin II inhibitors, (−) blebbistatin and Y-27632 is also significantly reduced. ***p < 0.001 for 0 hpi vs (−) blebbistatin or Y-27632.

To test if increase in RLC phosphorylation induced by viral infection modulates cellular stiffness, we measured the cellular stiffness of CHO cells using atomic force microscopy after SeV infection. Control CHO cells, which were not treated with SeV, exhibited a mean cortical stiffness of ~ 1 kPa (n > 200 cells). In contrast, CHO cells treated with SeV exhibited a complex temporal cortical stiffness response. Specifically, in line with increased RLC phosphorylation, at 2 hpi, cortical stiffness of SeV treated CHO cells increased dramatically from ~ 1 kPa to ~1.5 kPa (Fig. 7A). At 8 hpi, a mean cell stiffness of 1.2 kPa was observed, which was still higher compared to untreated cells. At 24 hpi, it was not possible to measure cell stiffness as cells were rounded and weakly adherent to the substrates. This could possibly be attributed to the onset of viral release (Fig. 3C,D). Cellular stiffness was also reduced when cells were treated with (−) blebbistatin or Y-27632 (Fig. 7A). Taken together with Figs. 3H,I and 4D, these data indicate that increased levels of NMII-A RLC phosphorylation (modulated by viral infection and contractility-altering drugs) positively correlate with increase in cell stiffness measurements.

## Discussion

Infection of host cells by paramyxoviruses comprises the following crucial events- binding, membrane fusion mediated entry and replication of its genome, followed by budding of the progeny virions^26^,^27^,^28^. Out of all these, the key step in successful viral infection involves complete fusion at plasma membrane level that in turn, encompasses two closely associated important processes- membrane mixing (hemifusion) and content mixing (formation of fusion pore)^29^. Although both these processes are known to be catalyzed by two viral spike glycoproteins viz. HN protein (for binding to host cell surface) and F protein (for membrane fusion), the fusion pore formation (previously attributed solely to the F protein) is currently known to be critically regulated by several host cell components. In fact, several studies on enveloped animal viruses have pointed out participation of various elements of the host cells for fine tuning the overall fusion process with special emphasis on the mechanistic of fusion pore formation^30^,^31^. We report that perturbing myosin II activity employing both pharmacological inhibitors and siRNA (Figs. 1, 2) increases viral fusion. Previous studies have demonstrated that during asymmetric cell division in *C. elegans,* localization of myosin II at anterior cell caused an increase in membrane tension, whereas a lack of myosin II at the posterior cell caused it to be comparatively relaxed^32^,^33^.It is pertinent to note that an increase in cell stiffness arises due to increase in levels of active Rho, which enhances myosin regulatory light chain phosphorylation. On the other hand, a decrease in myosin II levels or ROCK inhibition leads to cell softening and increase in viral fusion. Decisive evidence about the role of RLC phosphorylation has been provided by using a phospho-dead mutant of RLC (Ser-19) in modulating membrane fusion induced by Sendai virus (Fig. 6). This result along with Figs. 3 and 4D conforms to the notion of crucial involvement of Ser-19 of RLC of NMII-A in up-regulating membrane fusion and appears to be one of the major factors contributing to the novelty of this study. Phospho mimic mutant could not completely resist host cells from viral infection, and this may be due to insufficient replacement of endogenous RLC with the exogenous mutant form. Preparing knockout cells followed by expression of wild type and mutant RLC during viral infection may provide significant differences. Gerrits *et al*^21^ have shown that myosin II regulatory light chain has three isoforms and at this point little is known about the combination between light chain and heavy chain isoforms. Further study is warranted to establish the light chain isoform (s) specific knockout cell lines and the composition of NM II hexameric molecules. Increase in virus-host membrane fusion by inhibition of myosin activity correlates with our finding that reduction of cellular stiffness increases virus cell fusion which supports our hypothesis that membrane tension of the host cell built by the cortical actomyosin layer may create a barrier for viral fusion (illustrated in supplementary Fig.4). We can not rule out the possibility that dynamics of adhesome alter stiffness of the cell during viral infection. Studies are underway to decipher the correlation between dynamics of adhesome complex and viral infection.

We detected NMII-C in Huh7 cells using immunoblot analysis, and inhibited its expression by NMII-C specific siRNA. Interestingly, NMII-C knockdown Huh7 cells could not alter viral binding or membrane fusion (data not shown). Therefore, it appeared that NMII-C was less likely to be involved in membrane fusion. On the other hand, our data indicated that siRNA against NMII-B failed to alter the fusogenic activity of Sendai virus, suggesting thereby, that NMII-B was less likely to be involved during virus-cell fusion. Interestingly, NMII-B could act as fusion barrier during viral budding, as II-B knock down cells enhanced the release of viral particles as compared with II-A knock down or wild type cells. Recent report by Shutova *et al*^34^ shows that NM II exist as monomer in cytoplasm. Functional role of these monomer species is not known. Further studies are needed to establish whether NM II-B monomer can act as a viral particle transporter during budding. In contrast, II-A-knockdown cells exhibited more fusion with viral particles than the wild type cells, implying that NMII-A could act as fusion barrier during viral entry. Further investigation is underway to decipher the signaling pathways for regulating the differential role of NMII isoforms in viral entry and budding of Sendai virus life cycle.

Energy released due to conformational changes of viral F protein from metastatic prefusion to post fusion may couple with merging of two membranes^35^. On the other hand, conformational change of myosin II from 10S assembly-incompetent to 6S assembly-competent form due to light chain phosphorylation may provide a physical barrier by maintaining mechanical coherence of the cytoplasm^36^,^37^. This could be one of the ways the cell utilizes its own mechanism to resist viral fusion. Moreover, perturbation of Rho signaling has a more profound effect on membrane fusion than that of actomyosin dynamics. This leads us to hypothesize that Rho and some of its interacting partners are primarily associated with membrane fusion event in conjunction with actin and NMII. Interestingly, this conforms to the current hypothesis on membrane fusion that suggests as a pre-requisite to this event, the target membrane has to bend owing to its asymmetric nature^38^,^39^. Overall this phenomenon is assisted by differential binding of proteins to one of the membrane leaflets. Further studies are underway to find a molecular mechanistic correlation between cytoskeletal protein conformation and their direct or indirect involvement in membrane binding, which may regulate membrane fusion.

## Materials and Methods

### Virus propagation

Sendai virus (SeV, Z strain) was grown in embryonated chicken eggs and was purified according to standard procedures^40^. Two hemagglutinating units (HAU) were propagated in the allantoic sac of 10- to 11-day-old embryonated chicken egg. The viral yield was 200 HAU/ml from eight litres of allantoic fluid. Its fusogenic activity was assayed by standard hemolysis activity^41^. The amount of SeV was estimated by both Bradford reagent (Bio-Rad, Hercules, CA, USA) and hemagglutinin activity^42^.

### FHN-VLP preparation

F- HN VLP was prepared as previously described^40^. Briefly, 10^4 ^HAU SeV was solublilized with 5% Triton X-100 in TBS_7.4_. SM-2 biobeads were added (8 times the amt. of Triton X-100) to remove Triton X-100 according to previously published protocol^40^. The VLP suspension was taken out with the help of a 26 gauge needle avoiding biobeads after which it was centrifuged again to obtain the pellet which was resuspensed in mimimum volume of 1X PBS, pH 7.4. The fusogenic activity of F- HN VLP was assayed by hemolysis activity.

### Reagents

(−) blebbistatin (B0560), Y-27632 dihydrochloride (Y0503), Staurosporine (S3939, IC_50_ 20 nM)^43^,^44^, Chelerythrine (C2932, IC_50_ 1.77 μM)^23^, G_Ö_6976 (G1171)^24^,^45^, DMSO vehicle, protease inhibitors cocktail (P8340) and phosphatase inhibitor I and II cocktails (P2850 and P5726), neuraminidase (N2876), 4′, 6-diamidino-2 phenylindole (DAPI, D8417) Kodak-developer (P5670) and fixer (P6557), were purchased from Sigma-Aldrich. Octadecyl Rhodamine B Chloride (R18, O-246) and (+) blebbistatin (203392) were purchased from Life Technologies (Carlsbad, CA, USA) and EMD4 Biosciences, respectively.

### Cell culture, transfection and immunofluorescence microscopy

Chinese hamster ovary (CHO cells, CRL-9606) and Human hepatocarcinoma cells (Huh7 cells, JCRB0403) were obtained from American Type Culture Collection and Japanese Collection of Research Bioresources Cell bank, respectively, and maintained in Dulbecco modified Eagle medium (DMEM) supplemented with 10% fetal bovine serum (FBS) and antibiotics. For siRNA transfection, 66 pico mole siRNA was transfected to 3 × 10^5^ CHO or Huh7 cells using Lipofectamine^TM^ 2000 (Life Technologies). Transfection efficiency for siRNA (>90%) was estimated using a fluorescence microscope (Olympus IX-51) by visualizing the signal from Alexa 488 tagged nonspecific siRNA (Qiagen, Valencia, CA, USA). For immunostaining studies, CHO cells were grown on chamber slides (BD Bioscience, San Jose, CA, USA) and infected with SeV and at different time points (0-24 h) cells were washed with PBS and fixed with 4% paraformaldehyde at room temperature for 30 min, permeabilized with 0.5% Triton X-100 for 10 min, and treated with 5% normal goat serum (Santa Cruz Biotechnology, Dallas,TX, USA) for 60 min at room temperature followed by incubation with anti-NMHC II-A or II-B (Covance, Princeton, NJ, USA), and anti-F (Cosmo Bio Co. Ltd, Tokyo, Japan) antibodies overnight at 4 °C. The secondary antibody, Alexa fluor^®^ 488 goat anti-rabbit IgG and/or Alexa fluor^®^ 594 goat anti-mouse IgG (Life Technologies), was added at room temperature for 1 h. Nuclei was counterstained with DAPI. The images were collected using a Nikon C1 confocal microscope (Nikon, Tokyo, Japan). Line scans were used to analyze distribution of fluorescent probes. A line from one edge to another across the nucleus was drawn, and intensity was plotted against the distance using NIS-Element AR 4.2 software (Nikon).

### Atomic Force Microscopy

CHO cells were cultured on tissue culture plates for 24 h before initiating the experiments. Culture plates were mounted onto the stage of an Asylum MFP3D AFM (Asylum Research, CA) coupled to a Zeiss epifluorescence microscope and indented using a pyramid-tipped probe (Olympus) with nominal spring constant of 20 pN/nm. Actual spring constants were determined using thermal calibration method. Force curves were obtained for 30-40 cells for each condition and each time point. Force-indentation profiles were fit with a modified Hertzian model of a cone indenting a semi-infinite elastic material to extract the magnitude of cortical stiffness^46^.

### Neuraminidase treatment in CHO cells

CHO cells were infected with SeV at 30 MOI (multiplicity of infection) at 4 °C for 1 h. Unbound SeVs were washed with cold PBS (pH 7.4). To check if bound SeVs were attached through sialic acid containing receptor, neuraminidase (NA) was added at 220 μg/ml and kept the cells in 37 °C for 1 h. Cells were washed with cold PBS, and lysates were prepared by addition of Laemmli buffer. Lysates were subjected to 8% SDS-PAGE for immunoblot analysis with antibody specific for SeV proteins.

### Active Rho detection Assay

For the detection of active Rho in SeV treated CHO cells, biochemical affinity pull-down assay was carried out by using Active Rho-detection kit (Cell Signaling Technology, Danvers, MA) according to manufacturer protocol. Briefly, at 2 hpi with 30 MOI, CHO cells were washed with PBS and lysed with buffer (with 1 mM PMSF). Lysate was centrifuged at 16,000xg for 15 min at 4 °C. Approximately 5% of the supernatant was kept for total Rho detection and the rest was used for pull down assay. 500 μg of total protein from each sample was mixed with 400 μg GST-Rhotekin-RBD and 100 μl of 50% resin slurry in a spin column at 4 °C for 1 h. Column was washed with lysis buffer without PMSF, and bound active Rho was eluted with 2X sample buffer (with 200 mM DTT) was detected by immunoblot using antibody specific for Rho.

### Immunoblotting and immunoprecipitation

Extracts of SeV infected (MOI = 30) or noninfected CHO cells, which were pre-treated with drugs or siRNA, were prepared for SDS-PAGE as described previously^16^. Proteins were separated by SDS-PAGE on 8% or 12% polyacrylamide Tris-glycine gels, transferred to a polyvinylidene difluoride membrane (Millipore Corporation, Billerica, MA, USA), and blocked in 5% skim milk and 0.05% Tween 20 in phosphate-buffered saline, or 5% BSA and 0.05% Tween 20 in Tris buffered saline (pH 7.4). The upper part of the blot was incubated with antibodies to NMHC II-A or II-B (Cell Signaling Technology, Covance, USA) and lower part of the blot was incubated with antibodies to SeV (raised in rabbit), RLC, RLC-p (Cell Signaling) or GAPDH (Santa Cruz Biotechnology, Dallas, TX, USA) at 4°C overnight. Blots were then incubated with horseradish peroxidase-conjugated secondary antibodies against mouse or rabbit IgG (Sigma Aldrich or Thermo Fisher Scientific, Waltham, MA, USA) at room temperature for 1 h. Chemiluminescence signal was captured on Kodak film. Band intensity was quantified using ImageJ software (NIH, USA). For immunoprecipitation, extracts of SeV infected or noninfected CHO cells was prepared using an extraction buffer composed of 50 mM Tris-HCl (pH 8.0), 60 mM KCl, 10 mM MgCl_2_, 5 mM ATP, 4 mM EDTA, 1 mM dithiothreitol, 1% Nonidet P-40, 0.5 mM PMSF, and protease inhibitors cocktail and phosphatase inhibitor I and II cocktails at 4 °C. The lysates were sedimented at 10,000 x g for 10 min, and the supernatant was incubated with antibody specific to the NMHC II-A or II-B (Covance) as previously reported^47^. The immunoprecipitates were fractionated by SDS-PAGE on 8% or 12% polyacrylamide Tris-glycine gels, and subsequently, the blot was probed with antibodies specific to RLC, RLC-p or NMHC II. Protein concentrations were determined using a protein assay kit (Bio-Rad).

### Triton X-100 extraction of SV infected CHO cells

Triton X-100 soluble and insoluble fraction of SeV infected CHO cells were prepared as described^48^. In brief, CHO cells were infected with SeV at 30 MOI for different periods of time (0-24 h). At each time point, cells were washed and treated with buffer containing 50 mM Tris-HCl (pH 8.0), 5 mM NaCl, 140 mM Na-acetate, 0.6% Triton X-100, 5 mM EGTA, and 1 mM EDTA, protease and phosphatase inhibitors for 4 min at 4 °C. The extracted cell ghosts were scraped, and sheared through a 26-gauge syringe needle, and then centrifuged for 5 min at 4°C at 8000xg. The pelleted fraction is considered to contain the insoluble cytoskeleton, and the supernatant is the soluble portion. Both insoluble and soluble fractions were subjected to 10%SDS PAGE analysis.

### RT-PCR

Total RNA from SeV infected or uninfected CHO cells was isolated using the RNeasy mini kit (Qiagen, Venlo, Limburg, Netherland). 1 μg of total RNA was reverse transcribed using random hexamers and the Gene-Amp RNA PCR core kit (Life Technologies). The resulting cDNA was amplified by PCR using primers specific for NMHC II-A (702-944 nt), -II-B (3724-3958 nt) or GAPDH (730-955 nt) (Sequences were enlisted in supplemental Table 1). To check genomic DNA contamination in RNA samples, we performed cDNA synthesis in the absence of reverse transcriptase, which was used as a negative control for RT-PCR experiments. Products generated by RT-PCR were analyzed on a 1.8% agarose gel.

### Mutant constructs of RLC

A full-length mouse RLC cloned in pEGFP-C1 plasmid DNA was used as the template for PCR-based mutagenesis. Note that Serine 20 of mouse RLC is in equivalent position of Serine 19 of human RLC. One set of primer pair 5′- CTC AGC GCG CAA CCG CCA ATG TGT TCG CC-3′ and 5′- GGC GAA CAC ATT GGC GGT TGC GCG CTG AG -3′ were used for changing the codon from TCC (Ser 20) to GCC (Ala 20) and another set of primer pair 5′- CCC TCA GCG CGC AAC CGA CAA TGT GTT CGC CAT G -3 and 5′- CAT GGC GAA CAC ATT GTC GGT TGC GCG CTG AGG G -3 for TCC (Ser 20) to GAC (Asp 20). Resultant plasmid DNAs were sequenced to confirm the mutations. 1 μg of plasmid DNA was used to transfect 1 × 10^6^ cells using Lipofectamine^TM^ 2000. Since these constructs were introduced and expressed only transiently, the level of expression was assessed by immunoblot analysis using antibody against GFP. We normalized the transfection efficiency of all GFP-tagged RLC constructs to 75% of the cells by visualizing GFP signal under fluorescence microscopy.

### Mass spectroscopy

Abundance of each isoform of NMII in CHO cells was estimated by mass spectroscopy, as previously reported^49^,^50^. Briefly, the cell extract was fractionated by SDS-PAGE 6% polyacrylamide gels, and the gel was stained with Coomassie Blue. Bands near 220 kDa were excised followed by destaining, reduction and alkylation, and finally digestion with trypsin. Tryptic peptides were purified and concentrated on C18 resin Zip Tips (Millipore), and subjected to liquid chromatography tandem mass spectroscopy. Percentage contribution of each isoform to the total amount of NMHC II was calculated using the formula (n ⁄N) x 100 where n stands for total peptide numbers generated from an isoform and N is the total peptide numbers generated from all NMHC II isoforms.

### Kinetics of fusion of Sendai virus with CHO and Huh7 cells (lipid mixing)

Online fluorescence dequenching assay - purified SeV was labeled with R18 (Life Technologies) in 10 mM PBS (pH7.4) following previously published protocols^51^,^52^. In brief, 5 μg of R18 (prepared as 1 mg/ml in ethanol) was rapidly added to 100 HAU SeV in 1 ml PBS while vortexing and was then incubated in dark for 30 min. This was then passed through a Sephadex PD-10 column to remove free R18. The extent of R18 incorporation was calculated with respect to the fluorescence value obtained after addition of 1% Triton X-100. The activity of R18 labeled SeV was checked by standard hemolysis test, and it was found that majority of virion particles (more than 80%) remained fusogenically active. For the fusion kinetic study, ~1 × 10^6^ cells were treated with or without drugs for 1 h at 37 °C, or siRNA for 72 h at 37 °C. Then cells were lifted by 5 mM EDTA and after washing out EDTA, they were resuspended in PBS containing drugs/siRNA. R18 labeled SeV was added to cells at 30 MOI and kept at 4 °C for 40 mins for binding. Unbound SeVs were removed by low-speed centrifugation (300xg) for 4 mins at 4 °C. Virus-bound cells were resuspended in 100 μl of cold PBS, and added into a cuvette containing pre-warmed PBS under a continuous mixing condition by using a magnetic stir bar. Dequenching of R18-fluorescence (560 nm excitation and 590 nm emission) was monitored and fluorescence of R18 was recorded by a spectrofluorimeter (FL3-22, Horiba Jobin) at 37 °C. Change of fluorescence intensity, due to dequenching of R18, was calculated using an equation: %FDQ (Fluorescence Dequenching) = {(FT-F0)/(FTx-F0)}x100, where F0 and FT stand for fluorescence intensities at time zero and at a given time, FTx for fluorescence intensity in the presence of 0.1% Triton X-100, which is also defined as fluorescence at “infinite” dilution of probe (100%). In a similar way, siRNA treated cells were used for the dequenching assay with R18 labeled SeV.

### Statistical analysis

Data were expressed as the mean ± SEM. Student’s t-test was performed to check statistical significance in experimental data. The differences were considered to be significant if the p value was <0.05. Significance was shown as: * p < 0.05; ** p < 0.01; *** p < 0.001.

## Author Contributions

D.P.S. and S.S.J. conceived and directed the project, and edited the manuscript. P.D. carried out most of the experiments, analyzed the data and wrote the manuscript with input and contribution from all other co-authors - S.S., S.C., A.D., S.D., M.D. and Dr. S.S. 

## Additional Information

**How to cite this article**: Das, P. *et al*. Phosphorylation of Nonmuscle myosin II-A regulatory light chain resists Sendai virus fusion with host cells. *Sci. Rep.* **5**, 10395; doi: 10.1038/srep10395 (2015).

## Supplementary Material

Supplementary Information

Supplementary Information

Supplementary Information

## References

[b1] Dutch R. E. Entry and fusion of emerging paramyxoviruses. PLoS Pathog. 6, e1000881 (2010). 20585631 10.1371/journal.ppat.1000881 PMC2891828

[b2] Lamb R. A. & Jardetzky T. S. Structural basis of viral invasion: lessons from paramyxovirus F. Curr. Opin. Struct. Biol. 17, 427–436 (2007). 17870467 10.1016/j.sbi.2007.08.016 PMC2086805

[b3] Russell C. J. & Luque L. E. The structural basis of paramyxovirus invasion. Trends Microbiol. 14, 243–246 (2006). 16678421 10.1016/j.tim.2006.04.004 PMC7119026

[b4] Bissonnette M. L., Donald J. E., DeGrado W. F., Jardetzky T. S. & Lamb R. A. Functional analysis of the transmembrane domain in paramyxovirus F protein-mediated membrane fusion. J. Mol. Biol. 386, 14–36 (2009). 19121325 10.1016/j.jmb.2008.12.029 PMC2750892

[b5] Takimoto T., Taylor G. L., Connaris H. C., Crennell S. J. & Portner A. Role of the hemagglutinin-neuraminidase protein in the mechanism of paramyxovirus-cell membrane fusion. J. Virol. 76, 13028–13033 (2002). 12438628 10.1128/JVI.76.24.13028-13033.2002 PMC136693

[b6] Horvath C. M., Paterson R. G., Shaughnessy M. A., Wood R. & Lamb R. A. Biological activity of paramyxovirus fusion proteins: factors influencing formation of syncytia. J. Virol. 66, 4564–4569 (1992). 1602561 10.1128/jvi.66.7.4564-4569.1992 PMC241269

[b7] Duan R. & Gallagher P. J. Dependence of myoblast fusion on a cortical actin wall and nonmuscle myosin IIA. Dev. Biol. 325, 374–385 (2009). 19027000 10.1016/j.ydbio.2008.10.035 PMC2823627

[b8] Neco P. *et al.* Myosin II contributes to fusion pore expansion during exocytosis. J. Biol. Chem. 283, 10949–10957 (2008). 18283106 10.1074/jbc.M709058200

[b9] Rey M. *et al.* Myosin IIA is involved in the endocytosis of CXCR4 induced by SDF-1alpha. J. Cell Sci. 120, 1126–1133 (2007). 17327270 10.1242/jcs.03415

[b10] Tsai R. K. & Discher D. E. Inhibition of “self” engulfment through deactivation of myosin-II at the phagocytic synapse between human cells. J. Cell Biol. 180, 989–1003 (2008). 18332220 10.1083/jcb.200708043 PMC2265407

[b11] Dhawan J. & Helfman D. M. Modulation of acto-myosin contractility in skeletal muscle myoblasts uncouples growth arrest from differentiation. J. Cell Sci. 117, 3735–3748 (2004). 15252113 10.1242/jcs.01197

[b12] Swailes N. T., Colegrave M., Knight P. J. & Peckham M. Non-muscle myosins 2A and 2B drive changes in cell morphology that occur as myoblasts align and fuse. J. Cell Sci. 119, 3561–3570 (2006). 16895968 10.1242/jcs.03096

[b13] Berg J. S., Powell B. C. & Cheney R. E. A millennial myosin census. Mol. Biol. Cell 12, 780–794 (2001). 11294886 10.1091/mbc.12.4.780 PMC32266

[b14] Vicente-Manzanares M., Ma X., Adelstein R. S. & Horwitz A. R. Non-muscle myosin II takes centre stage in cell adhesion and migration. Nat. Rev. Mol. Cell Biol. 10, 778–790 (2009). 19851336 10.1038/nrm2786 PMC2834236

[b15] Dey S. K., Saha S., Das P., Das M. R. & Jana S. S. Regulation of nonmuscle myosin II during 3-methylcholanthrene induced dedifferentiation of C2C12 myotubes. Exp. Cell Res. 326, 68–77 (2014). 24887008 10.1016/j.yexcr.2014.05.015

[b16] Saha S. *et al.* The effect of including the C2 insert of nonmuscle myosin II-C on neuritogenesis. J. Biol. Chem. 288, 7815–7828 (2013). 23355468 10.1074/jbc.M112.417196 PMC3597820

[b17] Kovacs M., Toth J., Hetenyi C., Malnasi-Csizmadia A. & Sellers J. R. Mechanism of blebbistatin inhibition of myosin II. J. Biol. Chem. 279, 35557–35563 (2004). 15205456 10.1074/jbc.M405319200

[b18] Straight A. F. *et al.* Dissecting temporal and spatial control of cytokinesis with a myosin II Inhibitor. Science 299, 1743–1747 (2003). 12637748 10.1126/science.1081412

[b19] Sharma N. R. *et al.* Reciprocal regulation of AKT and MAP kinase dictates virus-host cell fusion. J. Virol. 84, 4366–4382 (2010). 20164223 10.1128/JVI.01940-09 PMC2863742

[b20] Betapudi V., Licate L. S. & Egelhoff T. T. Distinct roles of nonmuscle myosin II isoforms in the regulation of MDA-MB-231 breast cancer cell spreading and migration. Cancer Res. 66, 4725–4733 (2006). 16651425 10.1158/0008-5472.CAN-05-4236

[b21] Gerrits L. *et al.* Gene duplication and conversion events shaped three homologous, differentially expressed myosin regulatory light chain (MLC2) genes. Eur. J. Cell Biol. 91, 629–639 (2012). 22425609 10.1016/j.ejcb.2012.02.001

[b22] Dulyaninova N. G., Patskovsky Y. V. & Bresnick A. R. The N-terminus of the long MLCK induces a disruption in normal spindle morphology and metaphase arrest. J. Cell. Sci. 117, 1481–1493 (2004). 15020676 10.1242/jcs.00993

[b23] Tan I., Lai J., Yong J., Li S. F. & Leung T. Chelerythrine perturbs lamellar actomyosin filaments by selective inhibition of myotonic dystrophy kinase-related Cdc42-binding kinase. FEBS Lett. 585, 1260–1268 (2011). 21457715 10.1016/j.febslet.2011.03.054

[b24] Yang Q. *et al.* Protein kinase C activation decreases peripheral actin network density and increases central nonmuscle myosin II contractility in neuronal growth cones. Mol. Biol. Cell 24, 3097–3114 (2013). 23966465 10.1091/mbc.E13-05-0289 PMC3784383

[b25] Berdeaux R. L., Diaz B., Kim L. & Martin G. S. Active Rho is localized to podosomes induced by oncogenic Src and is required for their assembly and function. J. Cell Biol. 166, 317–323 (2004). 15289494 10.1083/jcb.200312168 PMC2172255

[b26] Cudmore S., Reckmann I. & Way M. Viral manipulations of the actin cytoskeleton. Trends Microbiol. 5, 142–148 (1997). 9141188 10.1016/S0966-842X(97)01011-1

[b27] Dohner K. & Sodeik B. The role of the cytoskeleton during viral infection. Curr Top Microbiol. Immunol. 285, 67–108 (2005). 15609501 10.1007/3-540-26764-6_3

[b28] Taylor M. P., Koyuncu O. O. & Enquist L. W. Subversion of the actin cytoskeleton during viral infection. Nat. Rev. Microbiol. 9, 427–439 (2011). 21522191 10.1038/nrmicro2574 PMC3229036

[b29] Chernomordik L. V. & Kozlov M. M. Membrane hemifusion: crossing a chasm in two leaps. Cell 123, 375–382 (2005). 16269330 10.1016/j.cell.2005.10.015

[b30] Laliberte J. P., Weisberg A. S. & Moss B. The membrane fusion step of vaccinia virus entry is cooperatively mediated by multiple viral proteins and host cell components. PLoS Pathog. 7, e1002446 (2011). 22194690 10.1371/journal.ppat.1002446 PMC3240603

[b31] Wurth M. A. *et al.* The actin cytoskeleton inhibits pore expansion during PIV5 fusion protein-promoted cell-cell fusion. Virology 404, 117–126 (2010). 20537366 10.1016/j.virol.2010.04.024 PMC2885465

[b32] Cabernard C., Prehoda K. E. & Doe C. Q. A spindle-independent cleavage furrow positioning pathway. Nature 467, 91–94 (2010). 20811457 10.1038/nature09334 PMC4028831

[b33] Ou G., Stuurman N., D’Ambrosio M. & Vale R. D. Polarized myosin produces unequal-size daughters during asymmetric cell division. Science 330, 677–680 (2010). 20929735 10.1126/science.1196112 PMC3032534

[b34] Shutova M. S., Spessott W. A., Giraudo C. G. & Svitkina T. Endogenous species of mammalian nonmuscle myosin IIA and IIB include activated monomers and heteropolymers. Curr. Biol. 24, 1958–1968 (2014). 25131674 10.1016/j.cub.2014.07.070 PMC4160463

[b35] Harrison S. C. Viral membrane fusion. Nat. Struct. Mol. Biol. 15, 690–698 (2008). 18596815 10.1038/nsmb.1456 PMC2517140

[b36] Cai Y. *et al.* Cytoskeletal coherence requires myosin-IIA contractility. J. Cell Sci. 123, 413–423 (2010). 20067993 10.1242/jcs.058297 PMC2816186

[b37] Sandquist J. C., Swenson K. I., Demali K. A., Burridge K. & Means A. R. Rho kinase differentially regulates phosphorylation of nonmuscle myosin II isoforms A and B during cell rounding and migration. J. Biol. Chem. 281, 35873–35883 (2006). 17020881 10.1074/jbc.M605343200

[b38] Boucrot E. *et al.* Membrane fission is promoted by insertion of amphipathic helices and is restricted by crescent BAR domains. Cell 149, 124–136 (2012). 22464325 10.1016/j.cell.2012.01.047 PMC3465558

[b39] Campelo F., McMahon H. T. & Kozlov M. M. The hydrophobic insertion mechanism of membrane curvature generation by proteins. Biophys. J. 95, 2325–2339 (2008). 18515373 10.1529/biophysj.108.133173 PMC2517036

[b40] Bagai S., Puri A., Blumenthal R. & Sarkar D. P. Hemagglutinin-neuraminidase enhances F protein-mediated membrane fusion of reconstituted Sendai virus envelopes with cells. J. Virol. 67, 3312–3318 (1993). 8388501 10.1128/jvi.67.6.3312-3318.1993 PMC237673

[b41] Peretz H., Toister Z., Laster Y. & Loyter A. Fusion of intact human erythrocytes and erythrocyte ghosts. J. Cell Biol. 63, 1–11 (1974). 4371393 10.1083/jcb.63.1.1 PMC2109346

[b42] Donald H. B. & Isaacs A. Counts of influenza virus particles. J. Gen. Microbiol. 10, 457–464 (1954). 13174769 10.1099/00221287-10-3-457

[b43] Wolf M. & Baggiolini M. The protein kinase inhibitor staurosporine, like phorbol esters, induces the association of protein kinase C with membranes. Biochemical and Biophysical Research Communications 154, 1273–1279 (1988). 3408497 10.1016/0006-291x(88)90277-x

[b44] Deng X., Ruvolo P., Carr B. & May W. S. Jr. Survival function of ERK1/2 as IL-3-activated, staurosporine-resistant Bcl2 kinases. Proc. Natl. Acad. Sci. USA 97, 1578–1583 (2000). 10677502 10.1073/pnas.97.4.1578 PMC26477

[b45] Mattoo A. R., Pastan I. & Fitzgerald D. Combination treatments with the PKC inhibitor, enzastaurin, enhance the cytotoxicity of the anti-mesothelin immunotoxin, SS1P. PLoS One 8, e75576 (2013). 24130723 10.1371/journal.pone.0075576 PMC3794001

[b46] Rotsch C., Jacobson K. & Radmacher M. Dimensional and mechanical dynamics of active and stable edges in motile fibroblasts investigated by using atomic force microscopy. Proc. Natl. Acad. Sci. USA 96, 921–926 (1999). 9927669 10.1073/pnas.96.3.921 PMC15326

[b47] Kim J. H. & Adelstein R. S. LPA(1) -induced migration requires nonmuscle myosin II light chain phosphorylation in breast cancer cells. J. Cell Physiol. 226, 2881–2893 (2011). 21302283 10.1002/jcp.22631 PMC3115449

[b48] Raab M. *et al.* Crawling from soft to stiff matrix polarizes the cytoskeleton and phosphoregulates myosin-II heavy chain. J. Cell Biol. 199, 669–683 (2012). 23128239 10.1083/jcb.201205056 PMC3494847

[b49] Babbin B. A. *et al.* Non-muscle myosin IIA differentially regulates intestinal epithelial cell restitution and matrix invasion. Am. J. Pathol. 174, 436–448 (2009). 19147824 10.2353/ajpath.2009.080171 PMC2630553

[b50] Ma X. *et al.* Ablation of nonmuscle myosin II-B and II-C reveals a role for nonmuscle myosin II in cardiac myocyte karyokinesis. Mol. Biol. Cell 21, 3952–3962 (2010). 20861308 10.1091/mbc.E10-04-0293 PMC2982113

[b51] Puri A., Clague M. J., Schoch C. & Blumenthal R. Kinetics of fusion of enveloped viruses with cells. Methods Enzymol. 220, 277–287 (1993). 8394493 10.1016/0076-6879(93)20089-l

[b52] Hoekstra D., de Boer T., Klappe K. & Wilschut J. Fluorescence method for measuring the kinetics of fusion between biological membranes. Biochemistry 23, 5675–5681 (1984). 6098295 10.1021/bi00319a002

