# Supplementary material for: Phosphorylation of Nonmuscle myosin II-A regulatory light chain resists Sendai virus fusion with host cells
Source: Sci Rep. 2015 May 20;5:10395. doi: 10.1038/srep10395 (PMC4438666; doi:10.1038/srep10395)
Supplement: Supplementary Information [file srep10395-s1.pdf]

Supplementary Information

Phosphorylation of Nonmuscle myosin II-A regulatory light chain resists Sendai virus fusion with host cells

Provas Das[1], Shekhar Saha[1], Sunandini Chandra[2], Alakesh Das[3], Sumit K. Dey[1], Mahua R Das[1], Shamik Sen[3], Debi P Sarkar[2*] and Siddhartha S Jana[1*]

Methods and Materials

Time-lapse microscopy of CHO cells infected with SeV. (-) bleb or vehicle treated CHO cells were infected with SeV at 30 MOI, and cells were monitored in a 37^0C and 5% CO2 stage incubator using a Nikon Ti-E microscope (Nikon, Tokyo). Images were recorded every 2 min for 12- 14 h using a CCD camera (Digital Sight DS-Qi1MC, Nikon) supported by the NIS-AR 4.2 program.

Immunostaining. NM IIs were visualized with Alexa-594 goat anti-rabbit. To stain actin filament, Alexa-488 tagged phalloidin (Life Technologies) was used.

Content mixing based on green and red fluorescent proteins (cell –cell fusion assay)

To assess complete fusion, we used a cell-cell fusion assay involving mixing of both leaflets of the plasma membranes and concomitant mixing of the aqueous contents of two types of cells-one mimicked virus and another mimicked host cells[1]. The one which mimicked virus was co-transfected with plasmids expressing HN, F and GFP and the second which mimicked host was transfected with plasmid expressing RFP using Lipofectamine™ 2000 (Life Technologies). Transfection efficiency was >75% as assessed by fluorescence microscopy. After 24h of transfection, viral mimicked cells were treated with 5µg/ml of trypsin and 0.22 mg/ml of neuraminidase for 20-30 mins at 37^0C. Host mimicking cells were treated with drugs for 1 h at 37^0C, lifted and replated on the top of neuraminidase treated viral mimicking cells and kept for 16-18h in CO_2 incubator at 37^0C. Images were captured with a digital camera (Digital sight DS-5M, Nikon) attached to a fluorescent microscope. Fused giant cells which showed yellow color in merging red and green channels using Image-Pro Plus Version 5.1 (Media

Cybernetics software package) were quantified by scoring the number of fused cells (yellow cells) in a given field as described earlier[2].

Movie 1 CHO cells treated with (-) bleb were infected with SeV and monitored for 12 h 37^0C humid incubator having 5% CO_2. Frames are 2mins apart and speed is 10 frames/second. Note that in presence of (-) bleb, SeV induces membrane mixing within12 hpi. Binucleated cells are visible due to (-) bleb treatment. hpi: hour post infection.

Movie 2 CHO cells treated with vehicle were infected with SeV and monitored for 12 h at 37^0C humid incubator having 5% CO_2. Frames are 2mins apart and speed is 10 frames/second.

Figure 1 (A) Cell-cell fusion by F and HN proteins was monitored by content mixing as described in Materials and Methods. Note that the yellow color in the merged image indicates complete fusion between green and red cells. Scale bar, 200 µm. More than 10 randomly chosen fields (n>100 cells in each sample) were used for quantification as shown in (B).

Figure 2 CHO cells infected with SeV for 2 h were stained with antibody to NMHC II-A (A, red), NMHC II-B (B, red) Alexa fluor® 488 tagged phalloidin (green) and DAPI (blue) to visualize NM II-A, NM II-B, actin filaments and nucleus, respectively. Images were captured using Zeiss LSM 710 confocal microscopy. Scale bar - 10 µm.

Figure 3 Lysates of CHO cells transfected with plasmid DNA containing WT-MLCK or GFP alone were probed with antibody against GFP, RLC-p, RLC, or GAPDH. Note that over expression of MLCK increases the level of RLCp.

Figure 4. Proposed model of Rho-ROCK dependent regulation of NMII-A RLC.
Left panel (A) shows that in the presence of virus, NMII-A is activated by Rho-ROCK pathway. Right panel (B) shows that perturbation of NMII-A activity induces disassembly of the actomyosin complex and consequently membrane fusion.

Table 1 Sequence of oligonucleotides:

Gene Name	Sequence (5'-3')	Product Size
NMHC IIA (MYH9)	Primer (Mouse)<br>(FP) CTCCTCACACAAGAGCAAGAAG<br>(RP) ATGTGGAAGGTCCGCTCCTCT	243 bp
	siRNA (Mouse)<br>(S) GUCAUCAACCCUUAUAAGA [dT][dT]<br>(AS) UCUUAUAAGGGUUGAUGAC [dT][dT]	
	siRNA (Human)<br>(S) GACAGAAUAGCUGAGUUCA [dT][dT]<br>(AS) UGAACUCAGCUAUUCUGUC [dT][dT]	
NMHC IIB (MYH10)	Primer (Mouse)<br>(FP) GGGACTTGAGTGAGGAGCTG<br>(RP) GCTTTGAACCTTTTCGCTTG	235 bp
	siRNA (Mouse)<br>(S) GCCAUAUCAGAGUCUGCUU [dT][dT]<br>(AS) AAGCAGACUCUGAUAUUGC [dT][dT]	
	siRNA (Human)<br>(S) GACAGAAUAGCUGAGUUCA [dT][dT]<br>(AS) UGAACUCAGCUAUUCUGUC [dT][dT]	
GAPDH	Primer (Mouse)<br>(FP) GACAACTTTGGCATTGTCGAA<br>(RP) ACACATTGGGGGTAGGAACA	226 bp

Reference

1. Sharma, N.R. *et al.* Reciprocal regulation of AKT and MAP kinase dictates virus-host cell fusion. *J Virol* **84**, 4366-4382 (2010)

2. Sha, Y. *et al.* A convenient cell fusion assay for the study of SARS-CoV entry and inhibition. *IUBMB Life* **58**, 480-486 (2006).

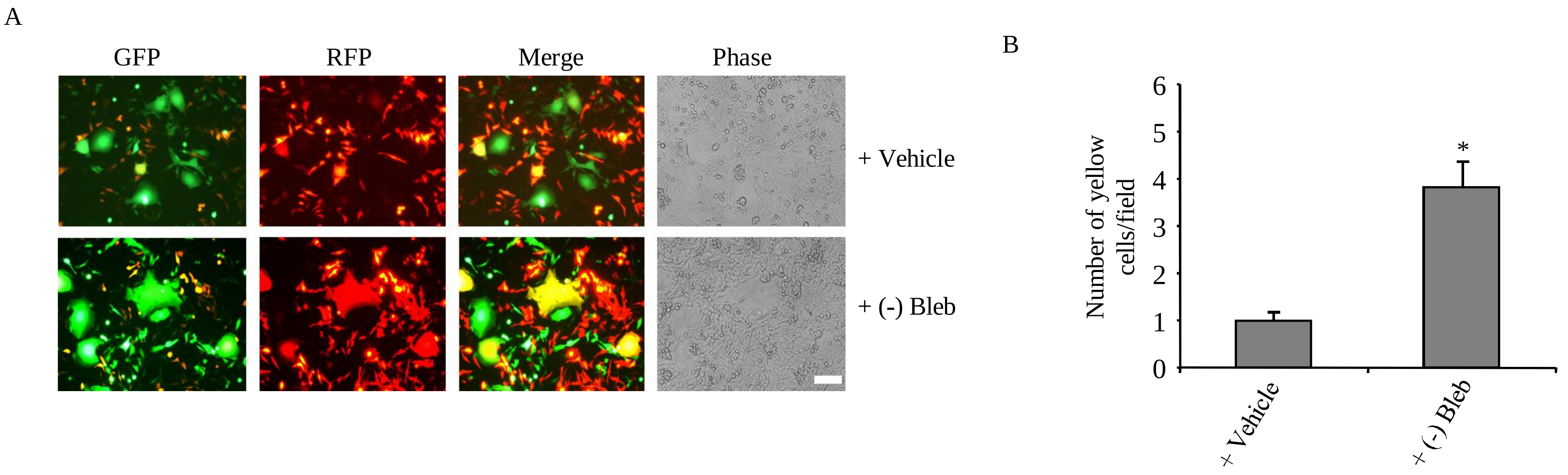
A
GFP
RFP
Merge
Phase
+ Vehicle
+ (-) Bleb
B
Number of yellow cells/field
0
1
2
3
4
5
6
*
+ Vehicle
+ (-) Bleb

Figure 1

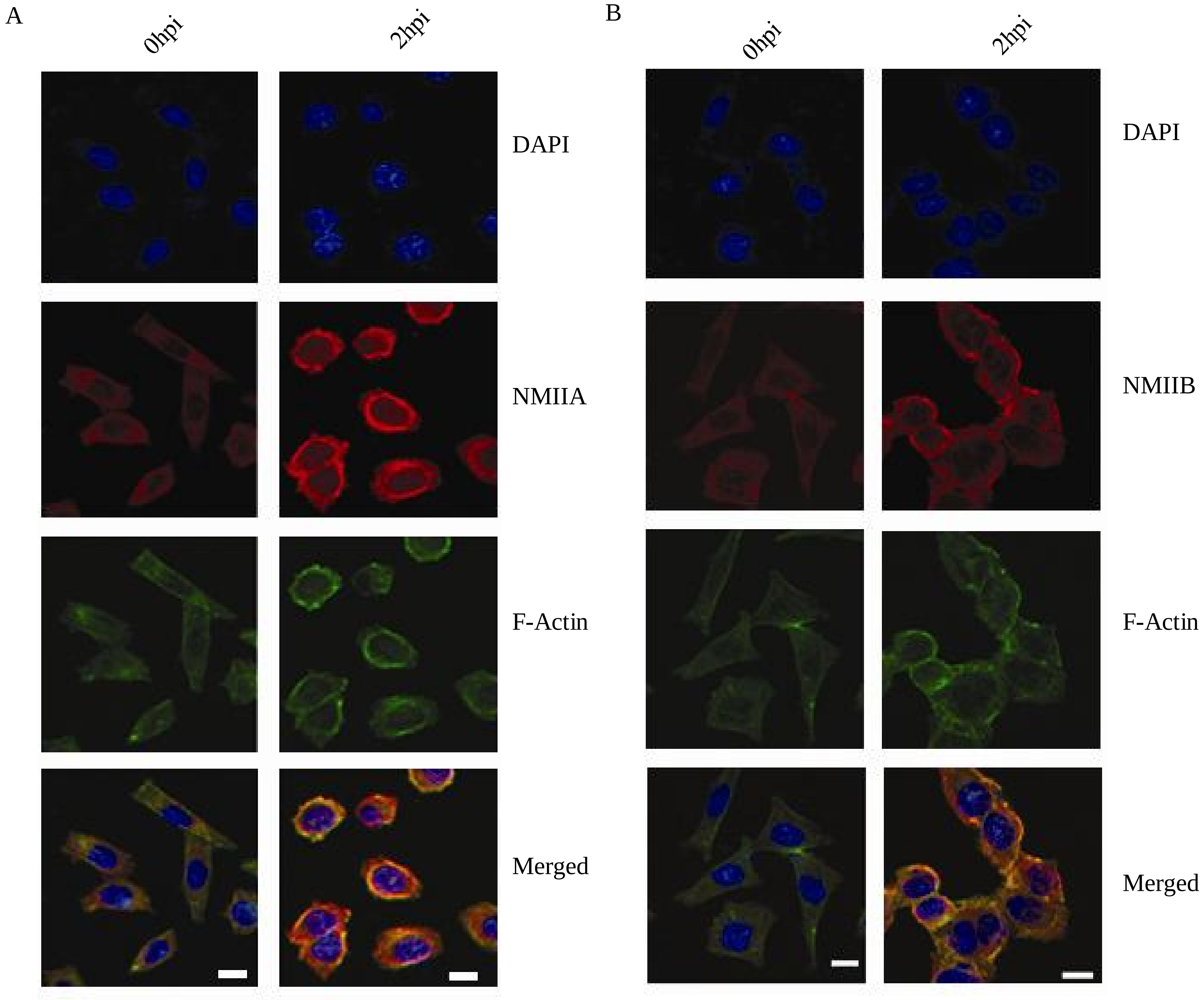
A
0hpi
2hpi
DAPI
NMIIA
F-Actin
Merged
B
0hpi
2hpi
DAPI
NMIIB
F-Actin
Merged

Figure 2

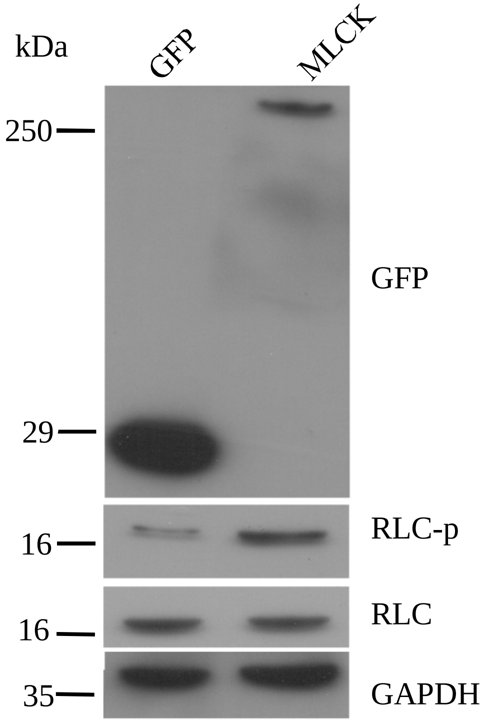

Figure 3

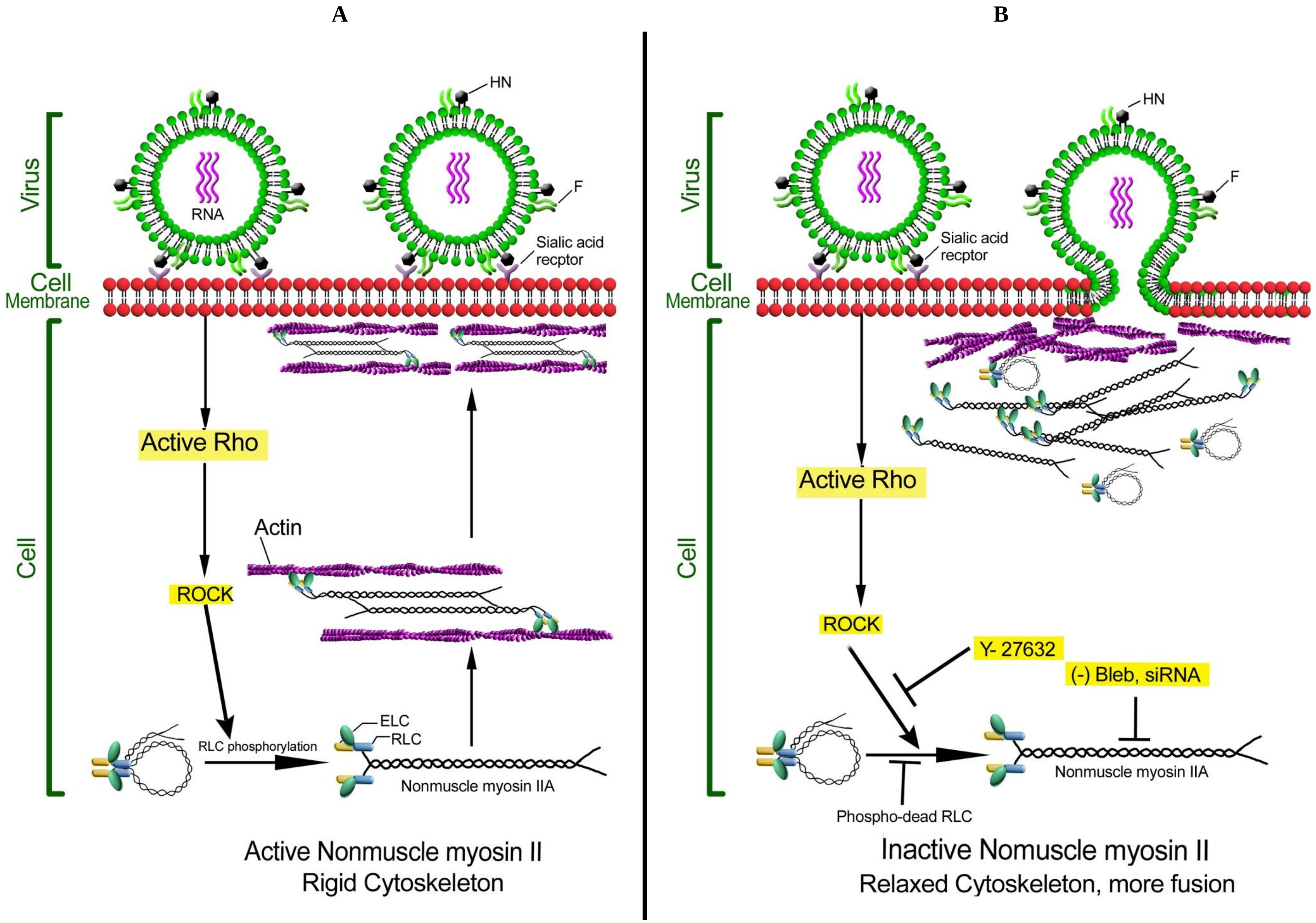
A
B
Virus
Cell
Membrane
Cell
HN
F
RNA
Sialic acid
recptor
Active Rho
ROCK
Actin
RLC phosphorylation
ELC
RLC
Nonmuscle myosin IIA
Active Nonmuscle myosin II
Rigid Cytoskeleton
Y- 27632
(-) Bleb, siRNA
Phospho-dead RLC
Inactive Nomuscle myosin II
Relaxed Cytoskeleton, more fusion

Figure 4
